# Cellular Senescence-Related Long Non-coding RNA Signatures Predict Prognosis in Juvenile Osteosarcoma

**DOI:** 10.1007/s43657-023-00132-y

**Published:** 2024-07-08

**Authors:** Peng Zhao, Junli Chang, YeKai Chen, Xingyuan Sun, Xiaoping Ma, Chujie Zhou, Lei Zhou, Yongjun Wang, Yanping Yang

**Affiliations:** 1 Longhua Hospital, Shanghai University of Traditional Chinese Medicine, Shanghai, 200032 China; 2 Key Laboratory of Theory and Therapy of Muscles and Bones, Ministry of Education, Shanghai, 200032 China; 3 Department of Oncology, Longhua Hospital, Shanghai University of Traditional Chinese Medicine, Shanghai, 200032 China

**Keywords:** Cellular senescence, Juvenile osteosarcoma, Long non-coding RNA, Prognostic model

## Abstract

Osteosarcoma is the most common malignant bone tumor and is frequently diagnosed in juvenile. Cellular senescence is a fundamental hallmark of osteosarcoma and plays a vital role in the initiation and progression of aging and tumorigenesis. Long non-coding RNAs (lncRNAs) are implicated in tumorigenesis. In this study, six cellular senescence-related lncRNAs with independent prognostic significance in juvenile osteosarcoma patients were identified through univariate Cox regression analysis, least absolute shrinkage and selection operator (LASSO) regression analysis, and multivariate Cox regression analysis. Prognostic significance was further confirmed by Kaplan–Meier (KM) survival curves, co-expression interaction networks, and sankey diagrams. A prognostic model of cellular senescence-related genes in juvenile osteosarcoma patients was then constructed using multivariate Cox regression analysis based on these six genes. High- and low-risk groups were identified according to the median risk score calculated by the prognostic model. The favorable prognostic significance of this model was demonstrated through survival curves, receiver operating characteristic (ROC) curves, distribution scatter plots and lncRNA expression heatmaps. Furthermore, cellular senescence-related lncRNAs were validated by enrichment analysis, immunological correlation analysis, m^6^A correlation analysis, and drug sensitivity correlation analysis. These findings are important for improving the prognosis of juvenile osteosarcoma patients and understanding the mechanisms underlying cellular senescence in juvenile osteosarcoma development.

## Introduction

Osteosarcoma is the most common primary malignant bone tumors, accounting for approximately 40% of primary bone malignancies (Gatta et al. 2017). The incidence rates of osteosarcoma is 0.21 of 100,000 per year, showing two age peaks of 60 years old and 15–19 years old (Gatta et al. 2017; Strauss et al. 2021). Based on a latest report by the American Cancer Society, the incidence rates for osteosarcoma are 4.3 of 100,000 per year at age 14 and younger, and 8.0 of 100,000 per year at age 15–19 (Siegel et al. 2023). Osteosarcoma is characterized by nocturnal pain, intermittent claudication and swelling, and even pathological fracture, which is concealed and easily confused in juvenile (Klein and Siegal 2006; Herman and Martinek 2015). Surgery is still the mainstay for osteosarcoma treatment (Marulanda et al. 2008). The systematic regimens of neoadjuvant chemotherapy combined with multidrug regimens and other adjuvant interventions have led to an increase in 5-year survival to about 70% in metastasis-free osteosarcoma patients since the 1980s (Corre et al. 2020). However, once metastasis or recurrence occurs, the overall 5-year survival rate of osteosarcoma patients is only 20% (Kansara et al. 2014). In the past four decades, it has been difficult to achieve significant breakthroughs in the diagnosis and treatment of osteosarcoma due to various reasons such as symptom limitations, metastasis, and challenges in developing new therapeutic targets (Allison et al. 2012).

Cellular senescence is a state that the cell cycle is permanently stalled in and limits the cell proliferative lifespan, which is one of the fundamental activities in cell growth and death, and presents in different physiopathological processes such as tissue remodeling, injury, cancer and senescence in the human body (Hayflick and Moorhead 1961; Muñoz-Espín and Serrano 2014; Sharpless and Sherr 2015; Hernandez-Segura et al. 2018). The cellular senescence can be traced back to the observations on cultured human diploid fibroblasts in the 1960s by Hayflick and Moorhead (1961) and has gradually emerged into the limelight in recent years as a basic phenotype of cellular senescence and neoplasia, however, it has not been adequately studied in juvenile diseases, such as osteosarcoma (Hanahan 2022; López-Otín et al. 2023). In addition to stable cell cycle arrest, cellular senescence is characterized by, firstly, increased cell size, flattened morphology, expanded mitochondrial and lysosomal networks, chromatin and nuclear rearrangements, and increased signature protein expressions, such as p53, p21 and p16 (Campisi and d'Adda di Fagagna 2007; Rai and Adams 2012; Burton and Krizhanovsky 2014). The second is the high senescence-associated β-galactosidase (SAβgal) activity (Dimri et al. 1995). The third is apoptosis resistance. Senescent cells (SNCs) display altered apoptotic signaling, often leading to resistance to programmed cell death (Childs et al. 2014; Burton and Faragher 2015). Fourthly, cellular senescence is accompanied by the release of many bioactive proteins (pro-inflammatory factors, chemokines, cytokines and proteases) and the senescence-associated secretory phenotype (SASP) (Childs et al. 2017; Roy et al. 2020). SASP plays a “double-edged” role at the initiation, establishment and escape of tumor cells (Pérez-Mancera et al. 2014), with senescence-associated secretory phenotypic factors promoting senescence induction in a paracrine manner, as well as the immune surveillance, leading to the elimination of senescent cells from cancerous and normal tissues (Krizhanovsky et al. 2008; Reimann et al. 2010; Kang et al. 2011). However, if not cleared in a timely manner, SASP exacerbates inflammation and pro-proliferative activity, thereby promoting tumorigenesis (Coppé et al. 2010).

According to the Human Genome Project, the encode genes only account for 1.5% of the human genome, and the rest belong to non-coding RNAs (ncRNAs), including long non-coding RNA (lncRNAs) (such as telomere RNA or TERRA (telomeric repeat-containing RNA)), microRNA (miRNAs) and circular RNAs (Fang et al. 2018; Uszczynska-Ratajczak et al. 2018). With the deepening of the research on lncRNAs, people gradually find that ncRNAs are not the “noise” of gene transcription, but play wide range of functions in various important biological processes. LncRNAs play important roles in cell proliferation and differentiation, gene expression regulation, RNA attenuation, RNA splicing, protein folding and miRNA regulation (Mattick et al. 2023). Moreover, it cannot be overlooked that lncRNAs play important roles in cancer development through interactions with other cellular macromolecules, including DNA, proteins and RNA, to drive many important cancer phenotypes (Winkle et al. 2021).

Based on the osteosarcoma clinical samples and cell lines, numerous unusually regulated lncRNAs with significant roles have been identified in multiple stages of osteosarcoma development. Meanwhile, miRNAs and lncRNAs have been found to be epigenetic factors with the ability to influence aging (Wz et al. 2022; López-Otín et al. 2023). However, there are few studies on the association between lncRNAs, cellular senescence and the occurrence and development of osteosarcoma.

Therefore, breaking through the diagnosis and treatment dilemma of juvenile osteosarcoma to achieve early diagnosis, metastasis prevention, and post-metastatic treatment for patients with bone tumors is the main directions of research. It is necessary to deeply understand the potential molecular mechanisms of bone cancers in juvenile from different perspectives for the development of innovative therapies to address the recurrence and metastasis, or enable early diagnosis.

## Materials and Methods

### Data Download and Collation

Sequencing and clinical data for juvenile (aged 10–19 years) with osteosarcoma were downloaded from the TCGA (The Cancer Genome Atlas) database (https://portal.gdc.cancer.gov/) on 17 December 2022, with the following search criteria set. In "Repository" screen, "data category" was set to "transcriptome profiling" and "data type" was set to "gene expression quantification", "workflow type" was set to "STAR-counts ", "program" was set to "TARGET-OS", "program" was set to "TARGET", "project" was set to "TARGET-OS", "Age at Diagnosis" was set to "Years-from 10 to 19" and "Vital Status" was set to "alive & dead".

The data downloaded from TCGA were further screened to eliminate any incorrect information that may have been missing or clinically inaccurate. In addition, gene IDs were matched and lncRNAs were identified using "vlookup" function of Microsoft Excel.

### Identification of Cellular Senescence Related lncRNAs in Juvenile Osteosarcoma

The set of cellular senescence-related genes was collected from Kyoto Encyclopedia of Genes and Genomes (KEGG) (https://www.kegg.jp/) and CellAge (https://genomics.senescence.info/cells/). The duplicates were removed using Microsoft Excel. Then the "limma" package of R language 4.1.2 was used to perform Pearson correlation analysis on cellular senescence-related lncRNAs in juvenile osteosarcoma, with a correlation coefficient of |R^2^|> 0.5 and *p* < 0.05 for identification.

### Prognostic Analysis of Cellular Senescence Related lncRNAs in Juvenile Osteosarcoma

Cellular senescence-related lncRNAs in juvenile osteosarcoma with prognostic significance were progressively accessed via the “survival”, “survminer” and “glmnet” package of R language 4.1.2 by the univariate Cox regression analysis, multivariate Cox regression analysis and Least absolute shrinkage and selection operator (LASSO) regression analysis. The cellular senescence-related lncRNAs with *p* < 0.05 in univariate Cox regression analysis were included in the LASSO regression analysis and multivariate Cox regression analysis.

Then, Cytoscape software 3.9.0 was used to visualize the co-expression network between prognostic lncRNAs and cellular senescence-related genes. Additionally, R language 4.1.2 was employed to produce Sankey diagrams in order to describe the relationship between risk types (high- or low-risk groups), prognostic lncRNAs, and cellular senescence-related genes.

### Establishment of Prognostic Model

By identifying the coefficient of cellular senescence-related lncRNAs with prognostic significance in juvenile osteosarcoma, prognostic models were established based on the following equation.

$${\text{Riskscore}} = \sum\limits_{{{\text{i}} = 1}}^{n} {\beta _{{\text{i}}} \times ({\text{expression}}\,{\text{of}}\,{\text{RNA}}_{{\text{i}}} )}$$

Then, as the risk scores of all the samples were calculated by this equation, the median risk score was obtained, and the high- and low-risk groups were divided out.

### Analysis of Prognostic Model

Based on the constructed prognostic model, all samples were described using R language 4.1.2 according to risk scores and risk groups for both the distribution characteristics and the relationships with gene expression.

Initially, The Kaplan–Meier (KM) survival curves were plotted to describe the difference in survival time between the high and low risk groups. The receiver operating characteristic (ROC) curves were plotted to describe the predictive effects of the model.

Scatter plots were generated by plotting samples with the risk score ranks from the smallest to the largest as horizontal coordinates, while the corresponding risk scores as vertical coordinates. The high- and low-risk groups were marked to characterize the distribution after the median risk scores were labeled. Subsequently, scatter plots were plotted with the risk score ranks (from the smallest to the largest) of samples as the horizontal coordinate, while survival time as the vertical coordinate to illustrate the relationship between risk score and survival time. Moreover, the heat maps, with high-/low-risk groups and the gene expression depicted by gradient colors, were constructed to represent the expression levels of the cellular senescence-related lncRNAs in juvenile osteosarcoma with prognostic significance in all samples, which further elucidates whether the cellular senescence-related lncRNAs with prognostic significance are differentially expressed in high- and low-risk groups. Finally, the survival Kaplan–Meier curve of cellular senescence-related lncRNAs with independent prognostic significance in juvenile osteosarcoma was plotted using the “survival” package in R language 4.1.2.

### Analysis of Clinical Characteristics

To determine the clinical characteristics data (including gender, metastasis, metastasis site, primary tumor site, specific tumor site, and risk score), the univariate and multivariate Cox regression analysis were conducted. The statistical analyses were performed using R language 4.1.2 with bilateral statistical tests and *p* < 0.05 as independent significance.

### Analysis of GO and KEGG Enrichment

Enrichment analysis of cellular senescence-associated lncRNA set was performed using the Gene Set Enrichment Analysis (GSEA) software 4.3.2. To visualize the Gene Ontology (GO) and KEGG pathways for cellular senescence-related genes, the researchers utilized the Gene sets database of "c5.go.v2022.1.Hs.symbols.gmt" and "c2.cp.kegg.v2022.1.Hs.symbols.gmt". The results of GSEA were subsequently arranged and presented in R language 4.1.2 using the "ggplot" package.

### Immunological Correlation Analysis

Firstly, Wilcoxon test in the "limma" package (Ritchie et al. 2015) of R language version 4.1.2 was used to assess the differences in expression levels of immune checkpoints between the high- and low-risk groups. Subsequently, the GSEA method was employed to analyze the immune function enrichment of the gene set, which was realized by the "GSVA" and "GSEABase" packages (Subramanian et al. 2005) in R language version 4.1.2. Finally, to estimate immune infiltration in juvenile osteosarcoma patients, Cell-type Identification By Estimating Relative Subsets of RNA Transcripts (CIBERSORT), Xcell, quantifying immune contexture of human tumors (quanTIseq), Tumor Immune Estimation Resource (TIMER), and Microenvironment Cell Populations-counter (MCP-counter) methods were utilized through TIMER 2.0 (http://timer.cistrome.org/) (Newman et al. 2015; Becht et al. 2016; Li et al. 2016; Aran et al. 2017; Finotello et al. 2019; Sturm et al. 2019). Subsequently, “pheatmap" package in R language 4.1.2 was used for the visualization of the heatmap.

### Analysis of Methylation-Related Genes

Differential expression of methylation-related genes in juvenile osteosarcoma patients between the high- and low-risk groups of juvenile osteosarcoma patients was demonstrated by the R language 4.1.2 with the package of “limma” (Ritchie et al. 2015).

### Drug Sensitivity Analysis

The efficacy profiles of 138 drugs, corresponding to more than 700 cell lines, were collected from the Cancer Genome Project (CGP) database (https://cancer.sanger.ac.uk/cosmic). The response to chemotherapy for each drug was predicted by using the "pRRophetic" package in R language version 4.1.2 (Geeleher et al. 2014) based on the RNA-seq and clinical data from juvenile osteosarcoma patients, along with the pharmacogenomic database mentioned above. The half-maximal inhibitory concentration (IC50) of each drug was estimated by ridge regression under all default parameters. The ComBat algorithm was used for all tissue types to eliminate any batch effects, and the duplicate gene expression data were combined by the averages, allowing the gene expression data to be standardized for the accuracy and reliability.

## Results

### A Total of 3972 Cellular Senescence-Related lncRNAs are Identified in Juvenile Osteosarcoma

Sixty-five patient samples were downloaded from TARGRT-OS, and 63 of them were finally included in this study. A total of 16,876 lncRNAs were screened from 60,660 genes, and 403 cellular senescence-related lncRNAs were collected from KEGG and CellAge. The duplicated lncRNAs were removed by excel. After Pearson correlation analysis, a total of 3972 lncRNAs with coefficient |R^2^|> 0.5 and *p* < 0.05 were finally identified as cellular senescence-related lncRNAs in juvenile osteosarcoma.

### Six Cellular Senescence-Related lncRNAs with Independent Prognostic Significance are Revealed

**Fig. 1:**
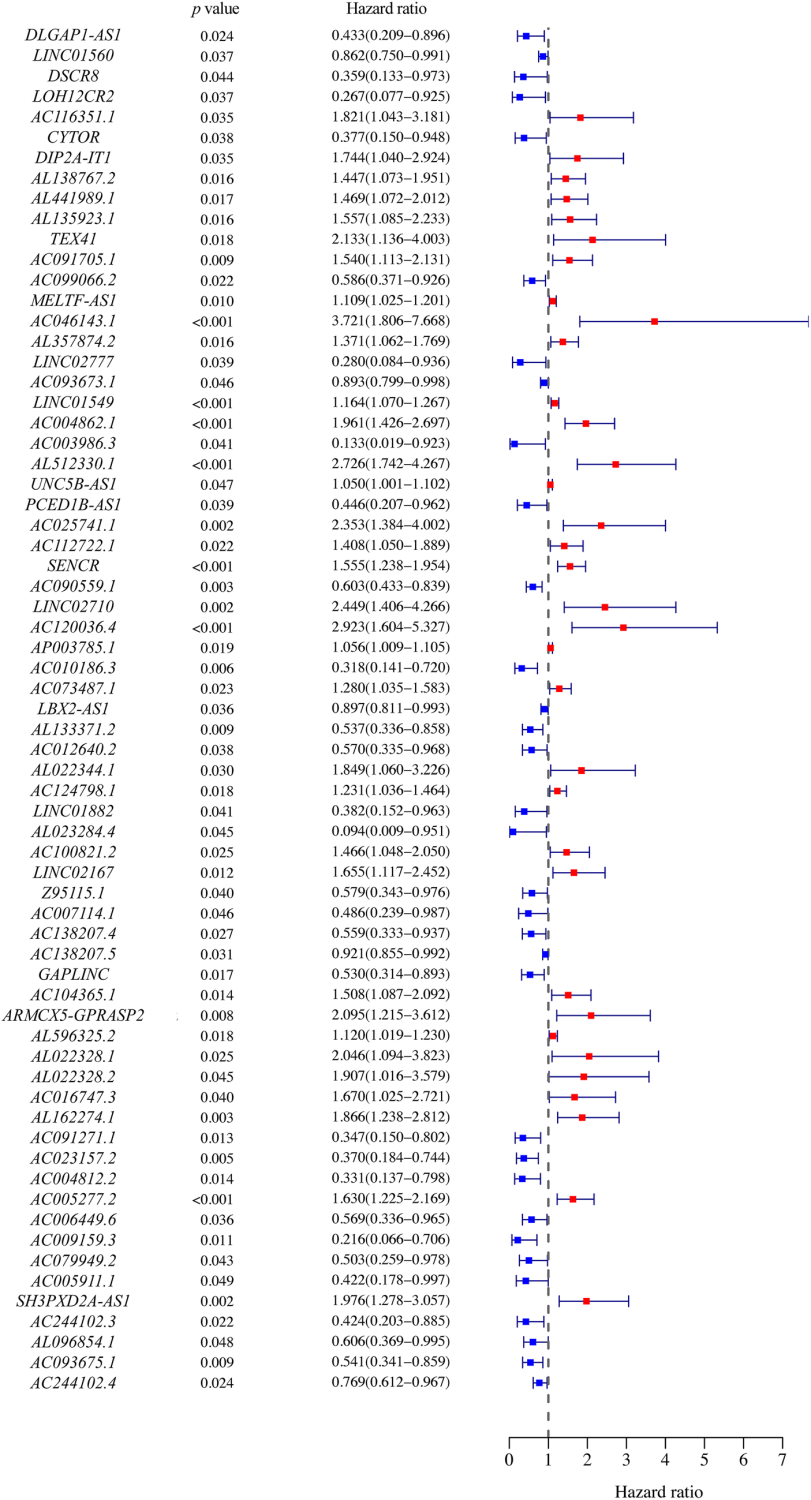
The forest map showing the hazard ratio and *p-*value of the 67 Cellular senescence-related lncRNAs with prognostic potential in juvenile osteosarcoma by univariate Cox regression analysis

**Fig. 2:**
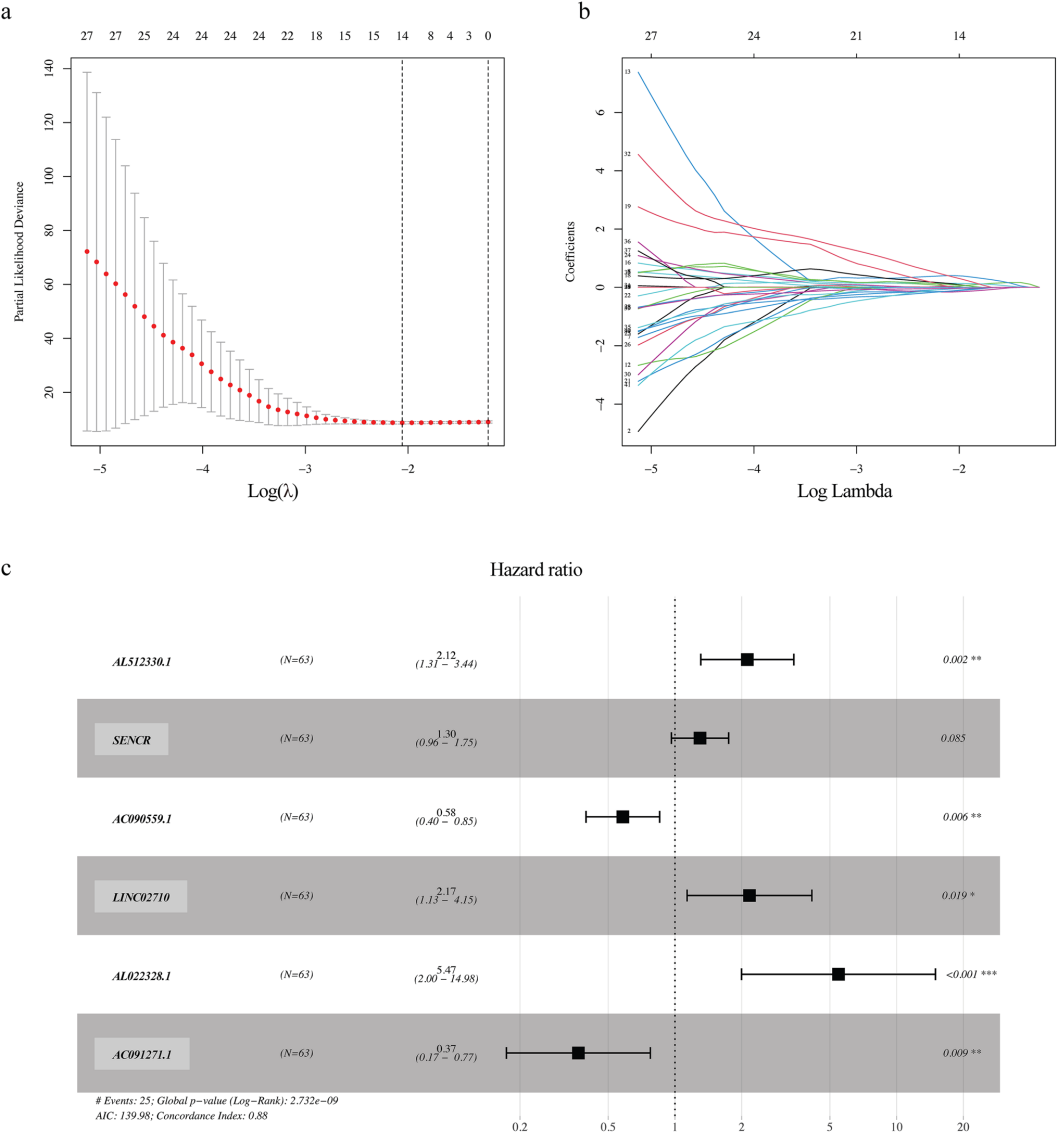
Screening the cellular senescence-related lncRNAs with independent prognostic significance in juvenile osteosarcoma by LASSO and multivariate Cox regression analysis. **a** LASSO coefficient values of six cellular senescence-related lncRNAs in juvenile osteosarcoma. The vertical dashed lines are the optimal log (lambda) value. **b** Profiles of LASSO coefficients. **c** The forest map showing the hazard ratio of the cellular senescence-related lncRNAs with independent prognostic significance of juvenile osteosarcoma by multivariate Cox regression analysis

Firstly, 67 cellular senescence-related lncRNAs were identified with prognostic value (*p* < 0.05) in juvenile patients by univariate Cox regression analysis (Fig. 1 and Table 1). Eleven cellular senescence-related lncRNAs with prognostic significance were further identified by LASSO regression analysis (Fig. 2a–b), which were finally included into the multivariate Cox regression analysis and six lncRNAs (*AL512330.1, SENCR (smooth muscle and endothelial cell enriched migration/differentiation-associated lncRNA), AC090559.1, LINC02710, AL022328.1 and AC091271.1*) were identified to be independent prognostic factors. Four lncRNAs (*AL512330.1, SENCR, LINC02710 and AL022328.1*) were deleterious prognostic factors and the others (*AC090559.1 and AC091271.1*) were favorable prognostic factors (Fig. 2c and Table 2).

**Table 1:** Significant prognostic value of cellular senescence-related lncRNAs for the survival of juvenile osteosarcoma patients by univariate Cox regression analysis

Gene name	KM	B	SE	HR	HR.95L	HR.95H	*p*-value
*DLGAP1-AS1*	0.00199253	– 0.8377839	0.3713097	0.4326683	0.20897565	0.89580703	0.02405222
*LINC01560*	0.03131901	– 0.1481589	0.0709195	0.86229404	0.75039269	0.99088254	0.03669743
*DSCR8*	0.00020611	– 1.023202	0.50808071	0.35944214	0.13278527	0.97298933	0.04402452
*LOH12CR2*	0.00432747	– 1.321803	0.63442908	0.2666541	0.0768991	0.92464553	0.03720998
*AC116351.1*	0.02227131	0.59946691	0.28454433	1.8211477	1.04265491	3.18089803	0.0351383
*CYTOR*	0.0003844	– 0.9767472	0.47121379	0.3765339	0.14952237	0.94820446	0.03818789
*DIP2A-IT1*	0.04638251	0.55630617	0.26362219	1.74421775	1.04041136	2.92412759	0.03483766
*AL138767.2*	0.01598281	0.36937488	0.15262966	1.44682988	1.07275085	1.95135405	0.0155174
*AL441989.1*	0.01456822	0.38439751	0.16049031	1.46872916	1.07233904	2.01164488	0.01661381
*AL135923.1*	0.00163621	0.44250799	0.18408789	1.55660628	1.08513279	2.23292776	0.01622624
*TEX41*	0.02979438	0.7573541	0.32130187	2.13262604	1.13611438	4.00320066	0.01841621
*AC091705.1*	0.02358264	0.4315519	0.16577952	1.53964504	1.11252256	2.13074947	0.00923668
*AC099066.2*	0.0481618	– 0.5341427	0.23303657	0.5861716	0.37124763	0.92552012	0.02189998
*MELTF-AS1*	0.01299078	0.10385442	0.04025481	1.10943893	1.02527047	1.20051711	0.00988215
*AC046143.1*	0.03627102	1.31401041	0.36891926	3.72106683	1.80568846	7.66817679	0.00036834
*AL357874.2*	0.04013298	0.31518697	0.13028716	1.37051553	1.06165481	1.76923121	0.01555591
*LINC02777*	0.00792368	– 1.2735464	0.61591974	0.27983745	0.08368238	0.93578843	0.03866685
*AC093673.1*	0.04290429	– 0.1132288	0.0566768	0.89294637	0.79906479	0.99785803	0.04573865
*LINC01549*	0.00612092	0.15200121	0.04324431	1.16416165	1.06955634	1.26713507	0.00043985
*AC004862.1*	0.00145039	0.67360196	0.16246064	1.9612891	1.42644446	2.69667348	3.38E-05
*AC003986.3*	0.01452584	– 2.0201484	0.98992957	0.13263578	0.01905597	0.92318825	0.04128074
*AL512330.1*	0.00717945	1.00280382	0.22857897	2.7259141	1.74158788	4.2665706	1.15E-05
*UNC5B-AS1*	0.04363825	0.04903026	0.02464687	1.05025213	1.00072354	1.10223203	0.04666702
*PCED1B-AS1*	0.01043652	– 0.8077959	0.39228298	0.44583966	0.20666495	0.96181285	0.03947342
*AC025741.1*	0.04067393	0.85576204	0.27095465	2.35316691	1.38361645	4.0021167	0.0015868
*AC112722.1*	0.00777359	0.3423306	0.14975356	1.40822578	1.0500303	1.88861201	0.02225661
*SENCR*	0.00166307	0.44148592	0.11644095	1.55501614	1.23771365	1.95366286	0.00014974
*AC090559.1*	0.00316577	– 0.5065469	0.16874326	0.60257272	0.4328874	0.83877212	0.00268318
*LINC02710*	0.04036749	0.89559527	0.28324787	2.44879305	1.4055654	4.26631689	0.00156755
*AC120036.4*	0.03611359	1.07251385	0.30624575	2.92271754	1.60365147	5.32676706	0.00046155
*AP003785.1*	0.01693592	0.05456423	0.02324487	1.0560803	1.0090458	1.10530722	0.01890663
*AC010186.3*	0.02003721	– 1.1449652	0.41667621	0.31823498	0.14062822	0.72015064	0.00599868
*AC073487.1*	0.03872497	0.2466318	0.10845622	1.27970784	1.03464825	1.58281054	0.02296468
*LBX2-AS1*	0.02337536	– 0.1086662	0.0516976	0.89702978	0.81059101	0.99268609	0.03555689
*AL133371.2*	3.17E-05	– 0.6215378	0.23885771	0.53711785	0.33632065	0.85779919	0.00926479
*AC012640.2*	0.00736137	– 0.5623053	0.27047536	0.5698938	0.33540141	0.96832908	0.03762177
*AL022344.1*	0.0273865	0.61462195	0.28395096	1.84895747	1.05980857	3.22571811	0.03042327
*AC124798.1*	0.02551593	0.20802884	0.08828599	1.23124868	1.03561089	1.46384451	0.01845768
*LINC01882*	0.04037529	– 0.9614305	0.47110979	0.38234555	0.15186114	0.96264335	0.04127289
*AL023284.4*	0.00205574	– 2.3626778	1.17988205	0.09416772	0.00932362	0.95108534	0.04523426
*AC100821.2*	0.00490795	0.38230056	0.17107859	1.46565253	1.04811433	2.04952578	0.02544042
*LINC02167*	0.02429659	0.50378113	0.20051839	1.65496711	1.11714053	2.45172032	0.01199152
*Z95115.1*	0.04900099	– 0.5465565	0.26641488	0.57893999	0.34344784	0.97590221	0.04021597
*AC007114.1*	0.00745002	– 0.7212987	0.36131096	0.48612053	0.23943935	0.98694377	0.04589718
*AC138207.4*	0.01404379	– 0.582065	0.26375718	0.55874334	0.33319758	0.93696394	0.02732652
*AC138207.5*	0.03708143	– 0.0819562	0.03793709	0.92131232	0.85529271	0.99242795	0.03074803
*GAPLINC*	0.00024679	– 0.6355013	0.26663157	0.52966991	0.31408565	0.89322836	0.01715152
*AC104365.1*	0.01272449	0.41092896	0.16689322	1.50821821	1.08743778	2.09181822	0.01380773
*ARMCX5-GPRASP2*	0.00358963	0.73964272	0.27783068	2.09518681	1.2154381	3.6117082	0.00776302
*AL596325.2*	0.02047036	0.11328551	0.04801338	1.11995164	1.01936615	1.23046237	0.0183017
*AL022328.1*	0.01314518	0.71568293	0.31912719	2.04558319	1.09439875	3.82347896	0.02492094
*AL022328.2*	0.02922194	0.64535544	0.32129318	1.90666461	1.0157552	3.57898236	0.0445776
*AC016747.3*	0.0126059	0.51262374	0.24911167	1.66966623	1.02467332	2.72065765	0.03960866
*AL162274.1*	0.01117698	0.62373909	0.20922656	1.86589174	1.23820476	2.81177404	0.00287153
*AC091271.1*	0.03501068	– 1.0579457	0.42740851	0.34716828	0.15022052	0.80232592	0.01331405
*AC023157.2*	0.04779451	– 0.9940724	0.3566043	0.37006655	0.18396606	0.74442673	0.0053099
*AC004812.2*	0.00090014	– 1.1065759	0.44951418	0.33068935	0.13702286	0.79808173	0.01382744
*AC005277.2*	0.03676807	0.48863565	0.14567187	1.63009069	1.22522443	2.16874197	0.00079549
*AC006449.6*	0.00689655	– 0.5636309	0.26917692	0.56913881	0.33581059	0.96458835	0.03626844
*AC009159.3*	0.02289537	– 1.5307254	0.60345189	0.21637864	0.06630636	0.70611195	0.01119298
*AC079949.2*	0.04080256	– 0.6863534	0.33900244	0.50340845	0.25903658	0.97831768	0.0429057
*AC005911.1*	0.02881279	– 0.8632086	0.43903174	0.4218065	0.17840572	0.9972815	0.04927962
*SH3PXD2A-AS1*	0.00293509	0.68117398	0.22258575	1.97619638	1.27751134	3.05699997	0.00221133
*AC244102.3*	0.01191215	– 0.8584887	0.37544964	0.42380209	0.20303915	0.88459892	0.02222143
*AL096854.1*	0.00406634	– 0.5005813	0.252981	0.60617816	0.36920066	0.99526356	0.04784631
*AC093675.1*	0.04962229	– 0.6140728	0.23568179	0.54114244	0.34095643	0.85886379	0.00917359
*AC244102.4*	0.00959553	– 0.2627915	0.11683328	0.7689022	0.61153657	0.96676246	0.02449429

**Table 2:** Significant prognostic value of cellular senescence-related lncRNAs for the survival of juvenile osteosarcoma patients by multivariate Cox regression analysis

ID	Coef	HR	HR.95L	HR.95H	*p-*value
*AL512330.1*	0.75153351	2.12024895	1.30817166	3.43644167	0.00228645
*SENCR*	0.2607126	1.29785461	0.9646685	1.74611961	0.08500935
*AC090559.1*	– 0.5412946	0.58199433	0.39687161	0.85346845	0.00558636
*LINC02710*	0.77427193	2.16901235	1.13419419	4.14797979	0.01925133
*AL022328.1*	1.6991026	5.4690373	1.99672912	14.9796828	0.00094947
*AC091271.1*	– 1.002949	0.36679618	0.17368977	0.77459619	0.00854772

**Fig. 3:**
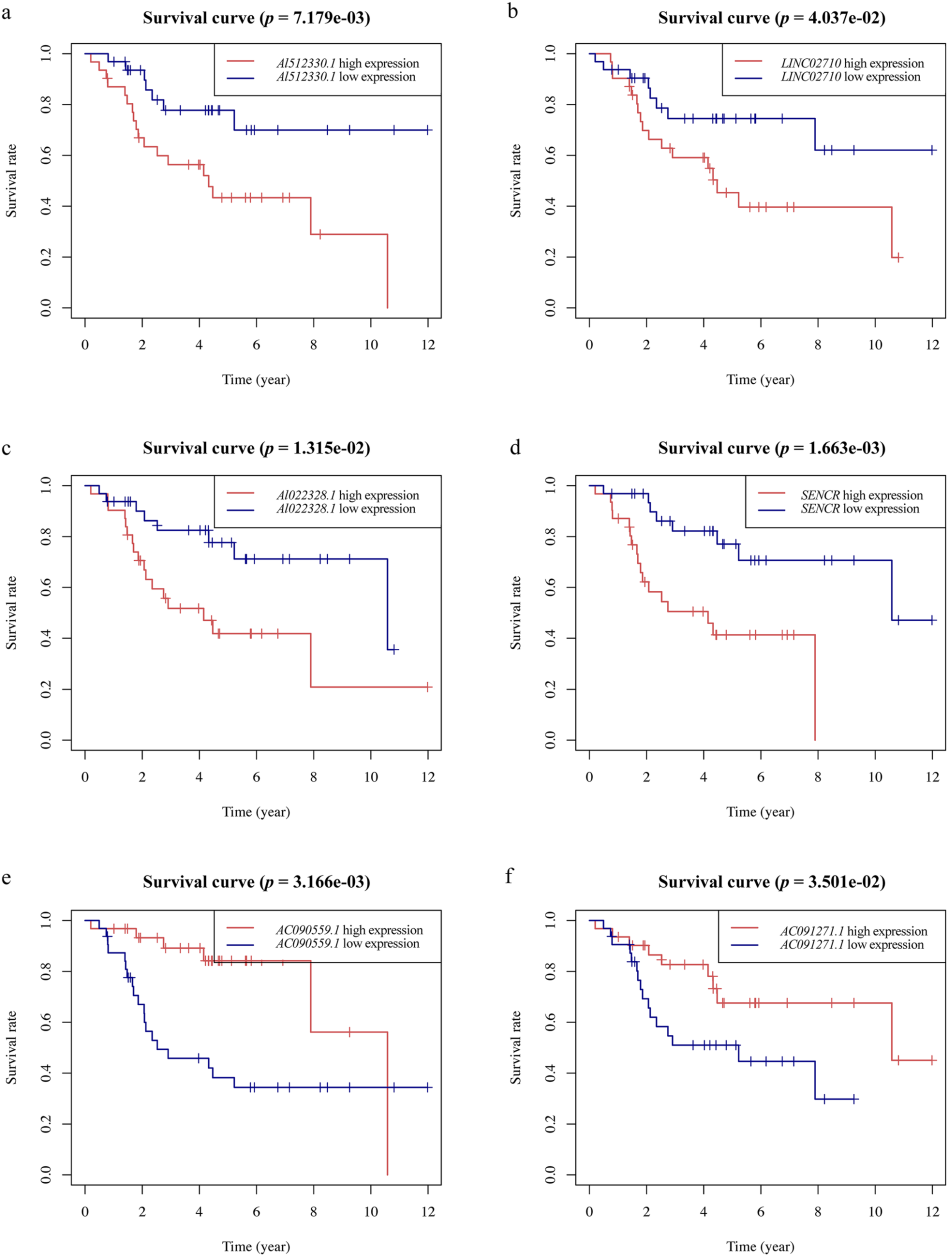
The KM survival curves of cellular senescence-related lncRNAs with independent prognostic significance in juvenile osteosarcoma (horizontal coordinates are survival times, vertical coordinates are survival rates, red and blue curves represent high and low expression of genes). Four lncRNAs (*AL512330.1, SENCR, LINC02710* and *AL022328.1*) with independent prognostic significance indicating the poor outcomes. Two lncRNAs (*AC090559.1* and *AC091271.1*) with independent prognostic significance indicating the favorable outcomes

The high and low expression levels of these six lncRNAs showed a significantly different survival in juvenile osteosarcoma patients determined by plotting the KM survival curves (Fig. 3). Patients with high expression levels of *AL512330.1, LINC02710, AL022328.1*, or *SENCR* showed lower survival times, while those with high expression levels of *AC090559.*1 or *AC091271.1* showed longer survival times. These findings suggest that the expression levels of these lncRNAs are correlated with the prognosis of juvenile osteosarcoma patients.

### Construction of a Cellular Senescence Prognostic Model for Juvenile Osteosarcoma Patients

The six cellular senescence-related lncRNAs with independent prognostic significance were further used to build a prognostic model for the juvenile osteosarcoma patients. The equation for the risk score was as follows: Risk Score = (0.75153351 * *AL512330.1*) + (0.2607126 * *SENCR*) + (0.77427193 * *LINC02710*) + (1.6991026 * *AL022328.1*)  –  (0.5412946 * *AC090559.1*) – (1.002949 * *AC091271.1*).

The median risk score of these 63 samples was calculated to be 0.5806584. Those samples with the risk score above the median were included in the high-risk group and the rest were included in the low-risk group.

**Fig. 4:**
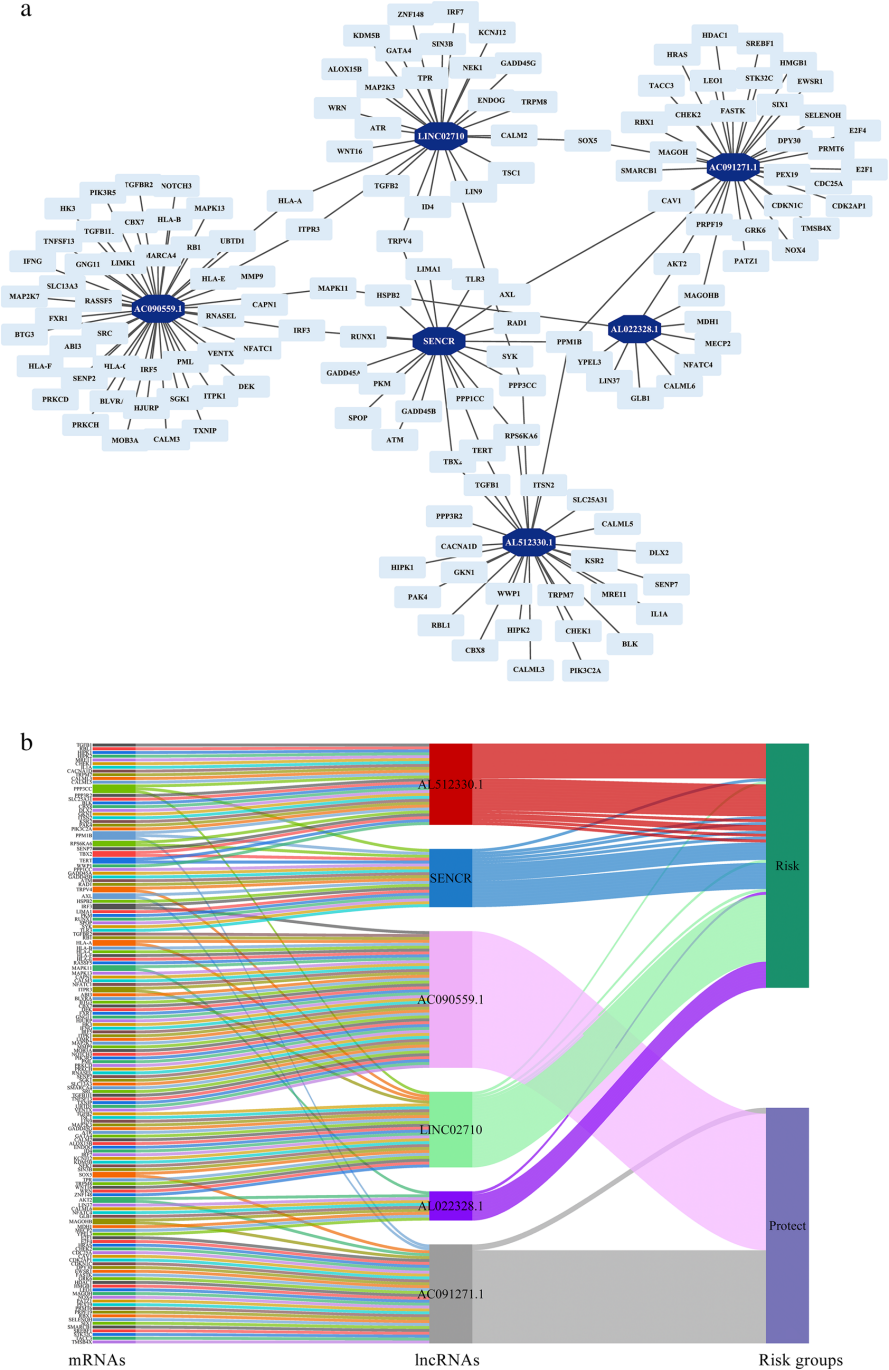
The coexpression network and Sankey diagram of cellular senescence-related lncRNAs with independent prognostic significance in juvenile osteosarcoma. **a** The coexpression network between cellular senescence-related lncRNAs with independent prognostic significance (dark blue nodes) and cellular senescence-related genes (light blue nodes) in juvenile osteosarcoma. **b** Sankey diagram showing the relationship between the cellular senescence-related lncRNAs with the independent prognostic significance, the cellular senescence-related genes or the risk groups

Finally, the relationship of the six prognostically independent cellular senescence-related lncRNAs and the risk types were depicted by co-expression network diagram and sankey plots (Fig. 4).

### The Prognostic Model of Cellular Senescence in Juvenile Osteosarcoma Patients Shows a Reliable Predictive Effect

**Fig. 5:**
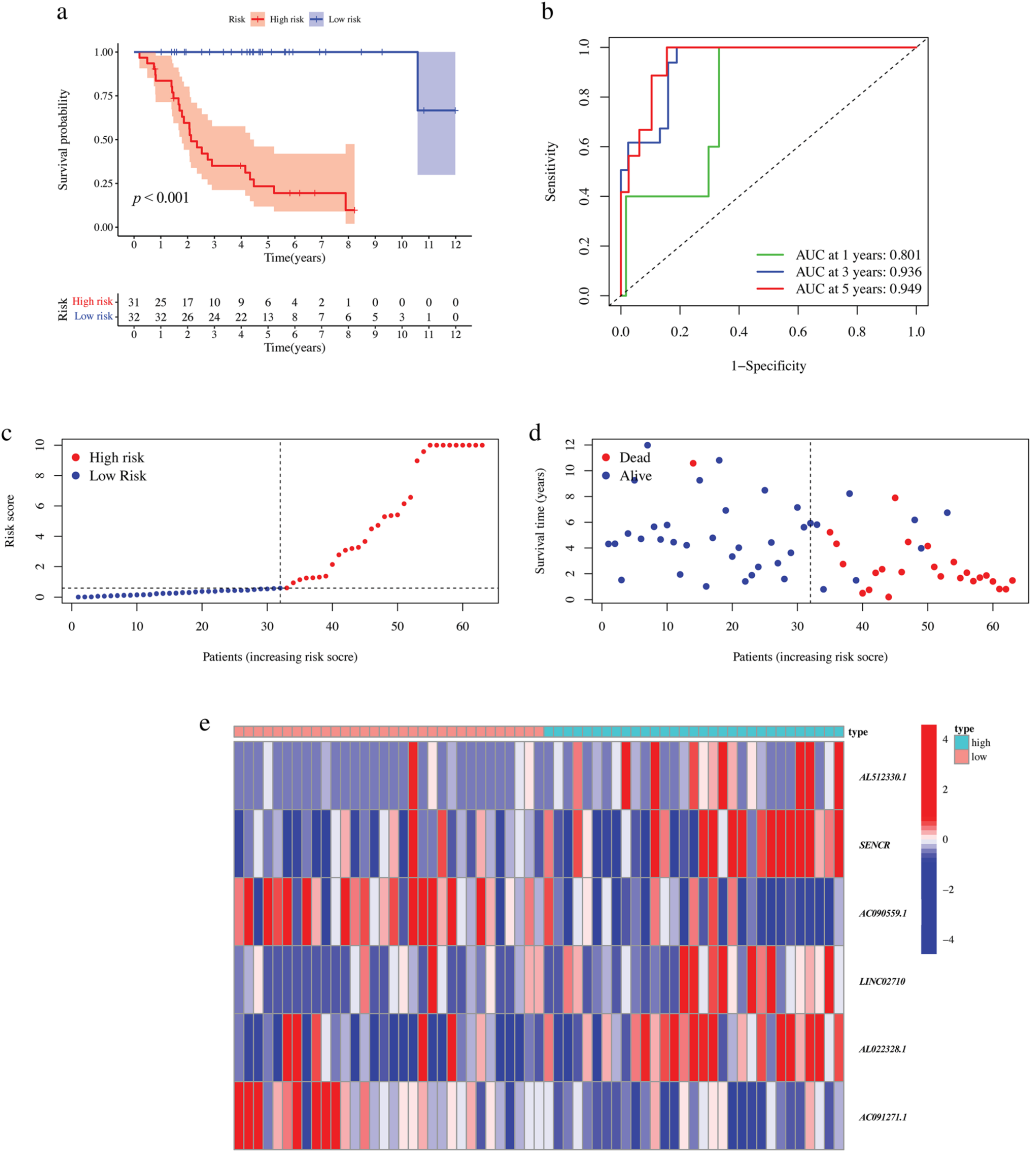
The prognostic model. **a** The KM survival curve of risk groups based on the risk score of prognostic model. **b** The ROC curve for prognostic model with 1-year, 3-year and 5-year AUC areas. **c** Distribution characteristics of risk score in the juvenile osteosarcoma sample with the increasing risk scores. **d** Distribution characteristics of survival time in the juvenile osteosarcoma sample with the increasing risk scores. **e** The expression heatmap of the six cellular senescence-related lncRNAs with independent prognostic significance in juvenile osteosarcoma sample

The KM survival curve analysis revealed that the survival time of juvenile osteosarcoma patients in the high-risk group was significantly lower than those in the low-risk group (*p* < 0.001, Fig. 5a).

As shown in Fig. 5c, all samples were sorted by risk score from the lowest to the highest. The scatter plots of survival time showed that patients with lower risk scores (low-risk group) had a trend to distribute in the upper part versus the patients with higher risk scores (high-risk group) (Fig. 5d), indicating that the low-risk group with lower risk scores has a longer survival time and the prognostic model is scientifically valid. The area under the ROC curve (AUC) is a commonly used method to determine the accuracy of a predictive model. A higher AUC indicates a better discrimination between the two groups. We found that the AUC was varied by year after the ROC curve was plotted for the prognostic model evaluation, with AUC of 0.801 for 1-year survival prediction, 0.936 for 3-year survival prediction, and 0.949 for 5-year survival prediction (Fig. 5b). These results suggest that the model has reliable predictive ability in identifying individuals who are at high risk of developing the disease within five years.

The heatmap illustrated the distinct expression patterns of the six lncRNAs in juvenile osteosarcoma samples, which were arranged according to their risk scores. *AC090559.1* and *AC091271.1* were found to be highly expressed in the low-risk group, while the remaining four lncRNAs (*AL512330.1*, *SENCR*, *LINC02710*, and *AL022328.1*) were highly expressed in the high-risk group (Fig. 5e). These findings reinforce the results of the aforementioned study.

**Fig. 6:**
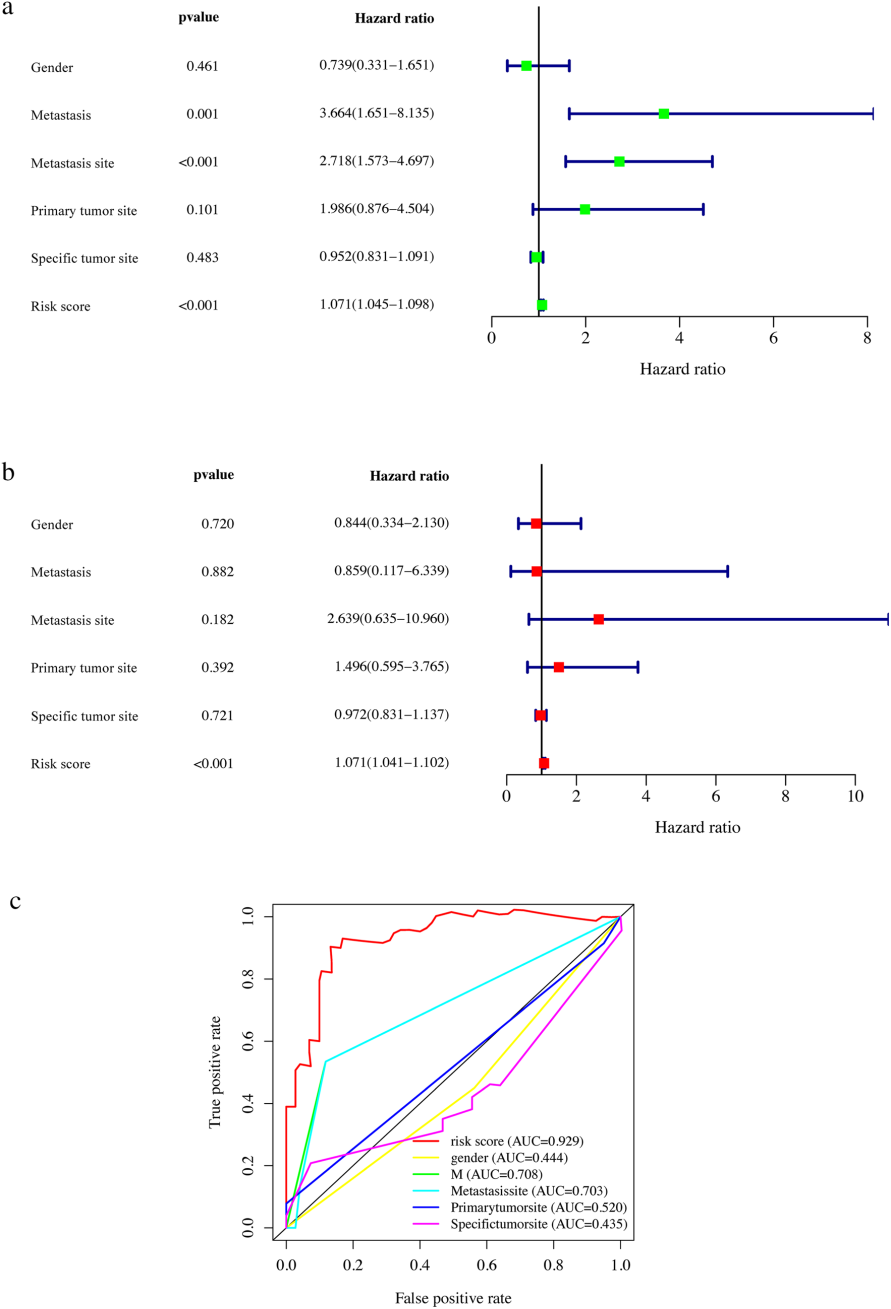
Clinical characteristics of juvenile osteosarcoma patients. **a** The forest plot for univariate Cox regression analysis of clinical characteristics. **b** The forest plot for multivariate Cox regression analysis of clinical characteristics. **c** The ROC curves for clinical characteristics

Age, metastasis, site of metastasis, primary site, site-specific and risk scores had been taken into account during the univariate and multivariate Cox regression analyses. The results of the univariate and multivariate Cox regression analyses revealed that the gender and the risk score of juvenile osteosarcoma were independent prognostic indicators. The hazard ratio of metastasis site was 2.718 (95% CI: 1.573–4.697, *p* < 0.001, Table 3, Fig. 6a), and the hazard ratio of risk score was 1.071 (95% CI: 1.045–1.098, *p* < 0.001, Table 3, Fig. 6a). After controlling the clinical characteristics, the risk score continues to be an independent prognostic indicator in the multivariate Cox regression analysis, with a hazard ratio of 1.071 (95% CI: 1.041–1.102, *p* < 0.001, Table 4, Fig. 6b). Furthermore, ROC curves for various predictors were plotted and the corresponding AUC values were obtained. The AUC regions for risk score, metastasis, and metastatic site were 0.929, 0.708 and 0.703, respectively, indicating that these predictors have good discriminatory power in identifying individuals at high risk to develop juvenile osteosarcoma. However, the AUC values for other predictors were all less than 0.5, suggesting that they may not be strong predictors for juvenile osteosarcoma (Fig. 6c).

**Table 3:** Clinical characteristics and risk scores of juvenile osteosarcoma using univariate Cox regression analysis

ID	B	SE	Z	HR	HR.95L	HR.95H	*p*-value
Gender	− 0.3026513	0.41013709	– 0.7379272	0.73885668	0.33071271	1.65070522	0.46055868
Metastasis	1.29860874	0.40690128	3.19145897	3.66419526	1.65053072	8.13455134	0.00141556
Metastasis site	0.99992736	0.27906286	3.58316176	2.71808438	1.57298362	4.6967957	0.00033946
Primary tumor site	0.68606389	0.41783809	1.64193717	1.98588347	0.8755669	4.50420537	0.10060303
Specific tumor site	− 0.0487048	0.06935327	– 0.7022712	0.95246225	0.83140793	1.09114227	0.48251004
Risk Score	0.06888259	0.01278488	5.38781606	1.07131042	1.04479917	1.09849438	7.13E-08

**Table 4:** Clinical characteristics and risk scores of juvenile osteosarcoma using multivariate Cox regression analysis

ID	B	SE	Z	HR	HR.95L	HR.95H	*p*-value
Gender	– 0.1696261	0.47234342	–0.359116	0.84398035	0.3344051	2.13005973	0.71950833
Metastasis	– 0.1514512	1.01948507	− 0.1485565	0.85945984	0.11653016	6.3388847	0.88190357
Metastasis site	0.97040395	0.72647836	1.33576442	2.63901028	0.63542102	10.9602532	0.18162628
Primary tumor site	0.40304354	0.47074666	0.8561793	1.49637205	0.59475665	3.76478232	0.39189861
Specific tumor site	– 0.0286246	0.0800696	– 0.3574963	0.97178121	0.83064055	1.13690419	0.72072029
Risk score	0.06890781	0.01447184	4.7615108	1.07133744	1.04137664	1.10216022	1.92E-06

**Fig. 7:**
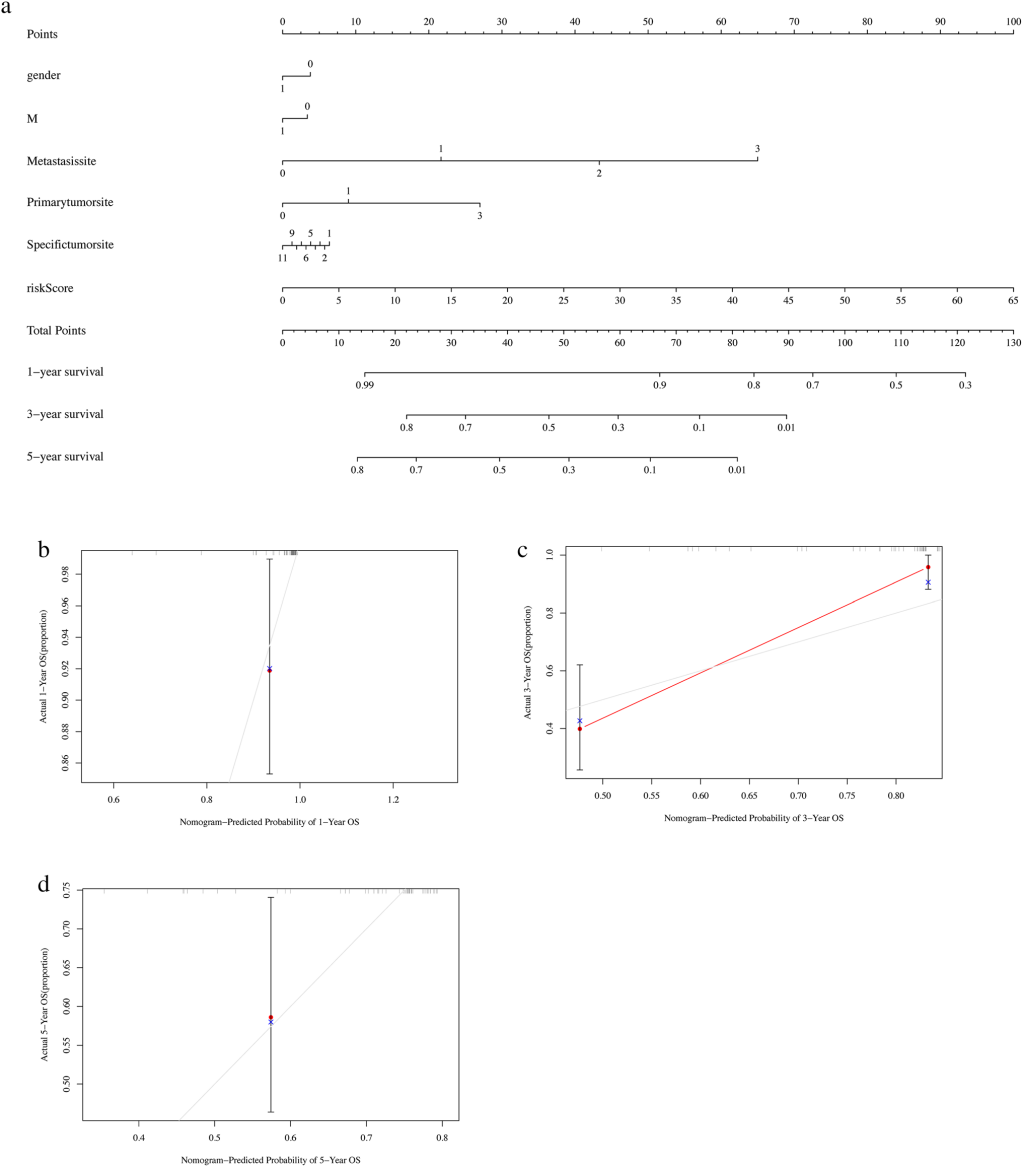
The assessment of prognostic models in juvenile osteosarcoma. **a** The nomogram of 1-year, 3-year or 5-year OS based on risk score, gender and metastasis. **b** Calibration plots evaluating the agreement between the predicted and the actual OS of 1-year for the prognosis model. The 45° reference line indicates perfect calibration, where the predicted probabilities are consistent with the actual probabilities. **c** Calibration plots evaluating the agreement between the predicted and the actual OS of 3-year for the prognosis model. **d** Calibration plots evaluating the agreement between the predicted and the actual OS of 5-year for the prognosis model

Finally, the nomograms of 1-year, 3-year or 5-year overall survival (OS) based on risk score, gender and metastasis were performed. As shown in the Norman plots, risk score and metastatic site showed the greatest impact on the survival at one, three and five years (Fig. 7a). And, the three-year prediction model had the best predictive efficiency as the calibration plots shown in Fig. 7b–d.

### Biological Pathways Associated with SASP Play Important Roles in the Development of Juvenile Osteosarcoma

Forty GO and KEGG pathways were obtained with GSEA software 4.3.2. After screening, four pathways (*p* < 0.05) were included in the summary curve using R language 4.1.2, respectively.

**Fig. 8:**
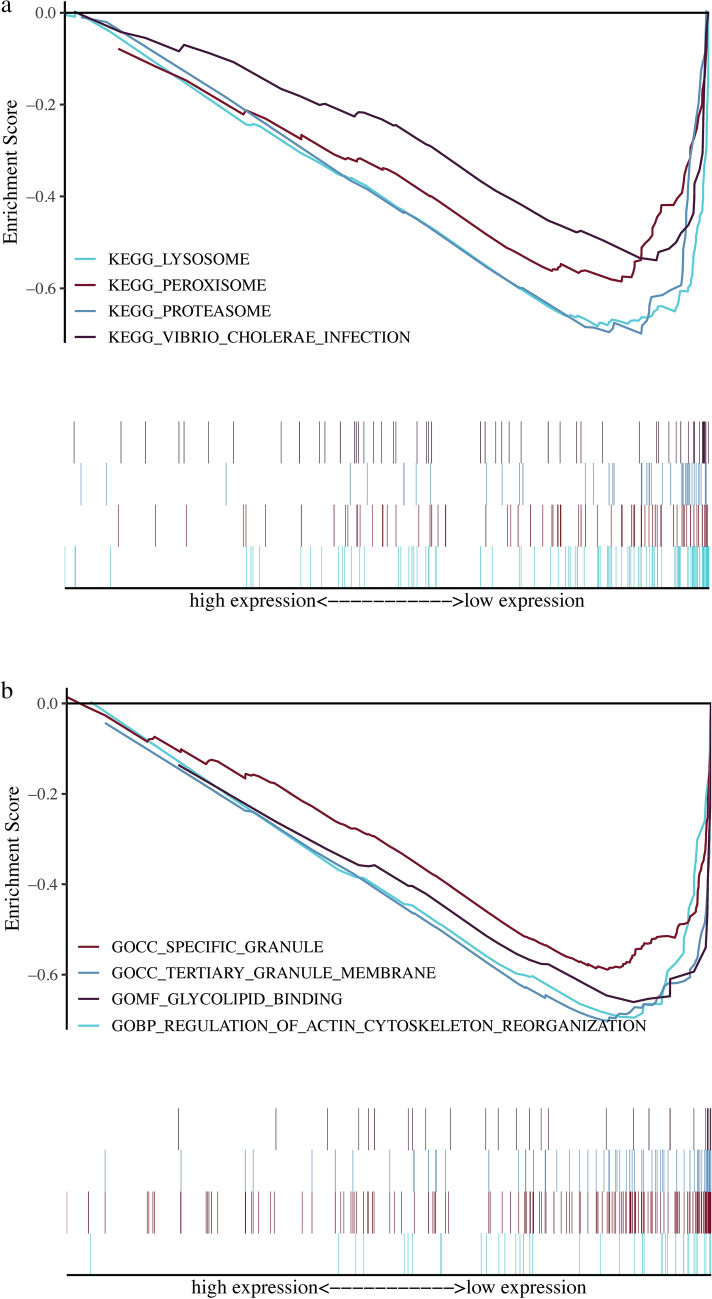
The curves for functional analysis based on cellular senescence-related lncRNAs. **a** KEGG enrichment analysis. **b** GO enrichment analysis

The cellular senescence-related lncRNAs in juvenile osteosarcoma focused on the lysosomes, the proteasomes, the peroxisomes, and the Vibrio cholerae infection based on the KEGG analysis (Fig. 8a) were mainly focused on regulating the actin cytoskeletal race pathway, the specific granules, the tertiary granule membranes, and the glycolipid binding based on the GO analysis, including molecular function (MF), cellular component (CC), and biological process (BP) (Fig. 8b).

### The Immune Cell Infiltration, Immune Cell Subsets and Checkpoint Inhibitors are Significantly Different in the High- and Low-Risk Groups

**Fig. 9:**
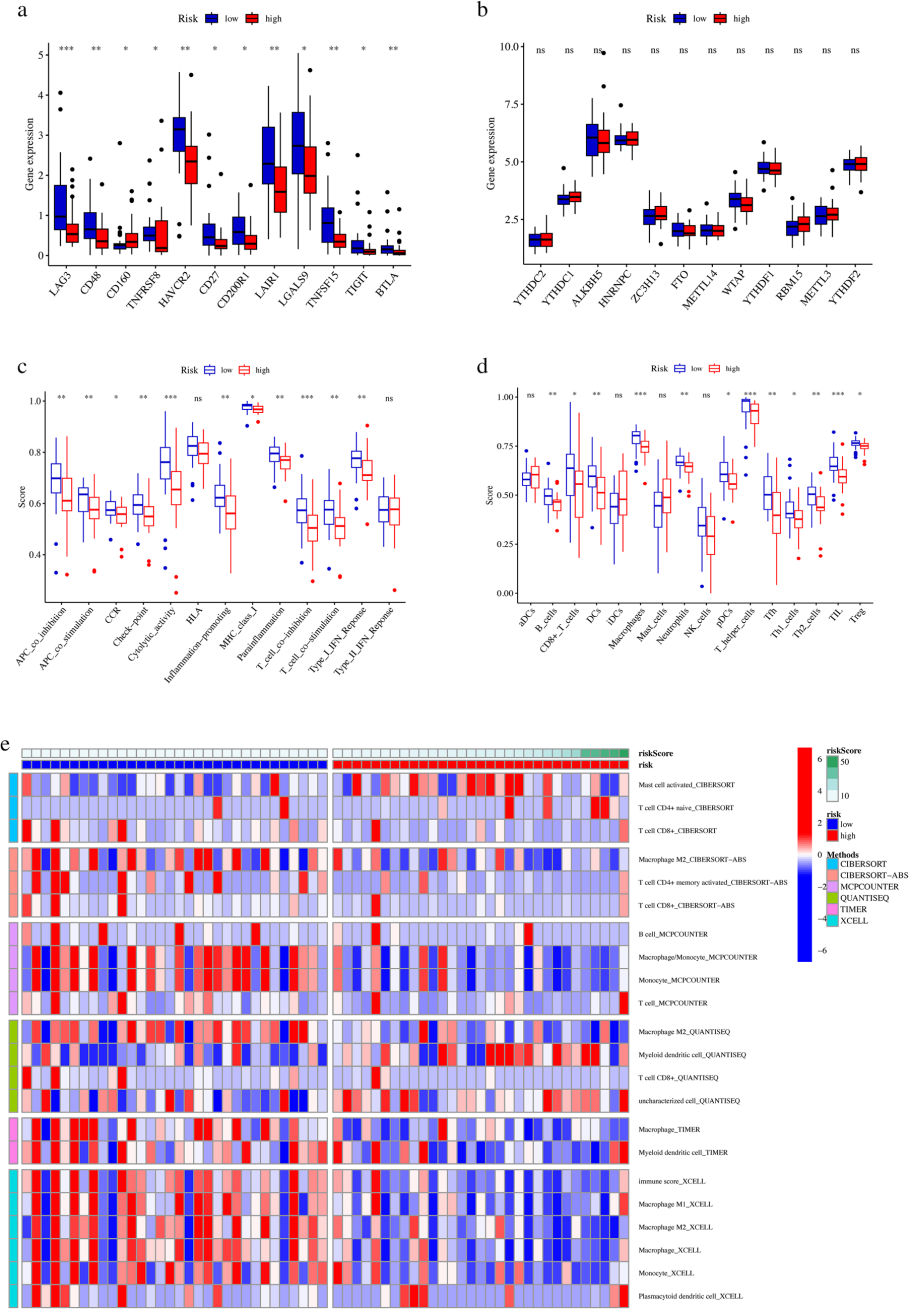
The immunological correlation and m^6^A-related gene analysis based on the cellular senescence-related lncRNAs with independent prognostic significance in juvenile osteosarcoma between the high- and low-risk groups. **a** The box plots of expression differences of immune check-point related genes in the high- and low-risk groups. **b** The box plots of expression differences of M^6^A related genes in the high- and low-risk groups. **c** The box plots of ssGSEA score differences of immune function enrichment in the high- and low-risk groups. **d** The box plots of ssGSEA score differences of immune cell enrichment in the high- and low-risk groups. **e** The heatmap of immune cell infiltration degree in all juvenile osteosarcoma samples ranked by risk score

Firstly, 47 immune checkpoints were included in this study, of which the 12 immune checkpoints (*LAG3(lymphocyte activating 3), CD48(CD48 molecule), CD160(CD160 molecule), TNFRSF8, HAVCR2, CD27, CD200R1(CD200 receptor 1), LAIR1(leukocyte associated immunoglobulin like receptor 1), TNFSF15(TNF superfamily member 15), TIGIT(T cell immunoreceptor with Ig and ITIM domains),* and *BTLA(B and T lymphocyte associated)*) were found to be differentially expressed in the high- and low-risk groups of juvenile osteosarcoma patients (*p* < 0.05). The expression levels of most immune checkpoints (except *CD160*) were higher in the low-risk group than in the high-risk group (Fig. 9a).

Furthermore, significant differences had been observed in the immune pathway scores (APC (Antigen-Presenting Cell) co-inhibition, APC co-stimulation, CCR (CC chemokine receptors), Check-point, cytolytic activity, Inflammation promoting, MHC (major histocompatibility complex) class I, Parainflammation, T cell co-inhibition, T cell co-stimulation, and Type I IFN Response) and the immune cell scores (B cells, CD8 + T cells, DCs (Dendritic cells), Macrophages, Neutrophils, pDCs, T helper cells, Tfh, Th1_cells, Th2_cells, TIL, and Treg) between these two groups. Additionally, ssGSEA scores for the above-mentioned differential immune pathways and cells were higher in the low-risk group than in the high-risk group, indicating that the juvenile osteosarcoma patients in the low-risk group may be more responsive to the immunotherapy (Fig. 9c–d).

To further explore the characteristics of immune cell infiltration between the high- and low-risk groups, the immune infiltration analysis by CIBERSORT, Cell-type Identification By Estimating Relative Subsets of RNA Transcripts with Absolute Quantities (CIBERSORT-ABS), MCP-counter, quanTIseq, TIMER, and Xcell methods were conducted. The CIBERSORT method identified that more activated mast cells were enriched in the high risk group, however, the enrichment of others (naive CD4^+^ T cells, CD8^+^ T cells) were not significant. The CIBERSORT-ABS method identified that more memory CD4^+^ T cells and CD8^+^ T cells were enriched in the low-risk group, but the enrichment of others (CD8^+^ T cells) were not significant. The MCP-counter method identified that more Macrophages / Monocyte and Monocyte were enriched in the low-risk group, while the enrichment of others (B cell and T cell) were not significant. The quanTIseq method identified that the M2 macrophages were more abundant in the low-risk group, and the myeloid dendritic cells were more abundant in the high-risk group, while the enrichment of others (CD8^+^ T cells and uncharacterized cells) were not significant. The TIMER method identified that both the macrophages and the myeloid dendritic cells were more abundant in the low-risk group. The Xcell method identified that macrophages, M1 macrophages, M2 macrophage and monocytes were more enriched in the low-risk group, while the enrichment of the plasmacytoid dendritic cells were not significant (Fig. 9e). And m^6^A related genes did not differ significantly between the high- and low-risk groups of juvenile osteosarcoma patients (Fig. 9b).

### Six Drugs Differ in Efficacy Between the High- and Low-Risk Groups

**Fig. 10:**
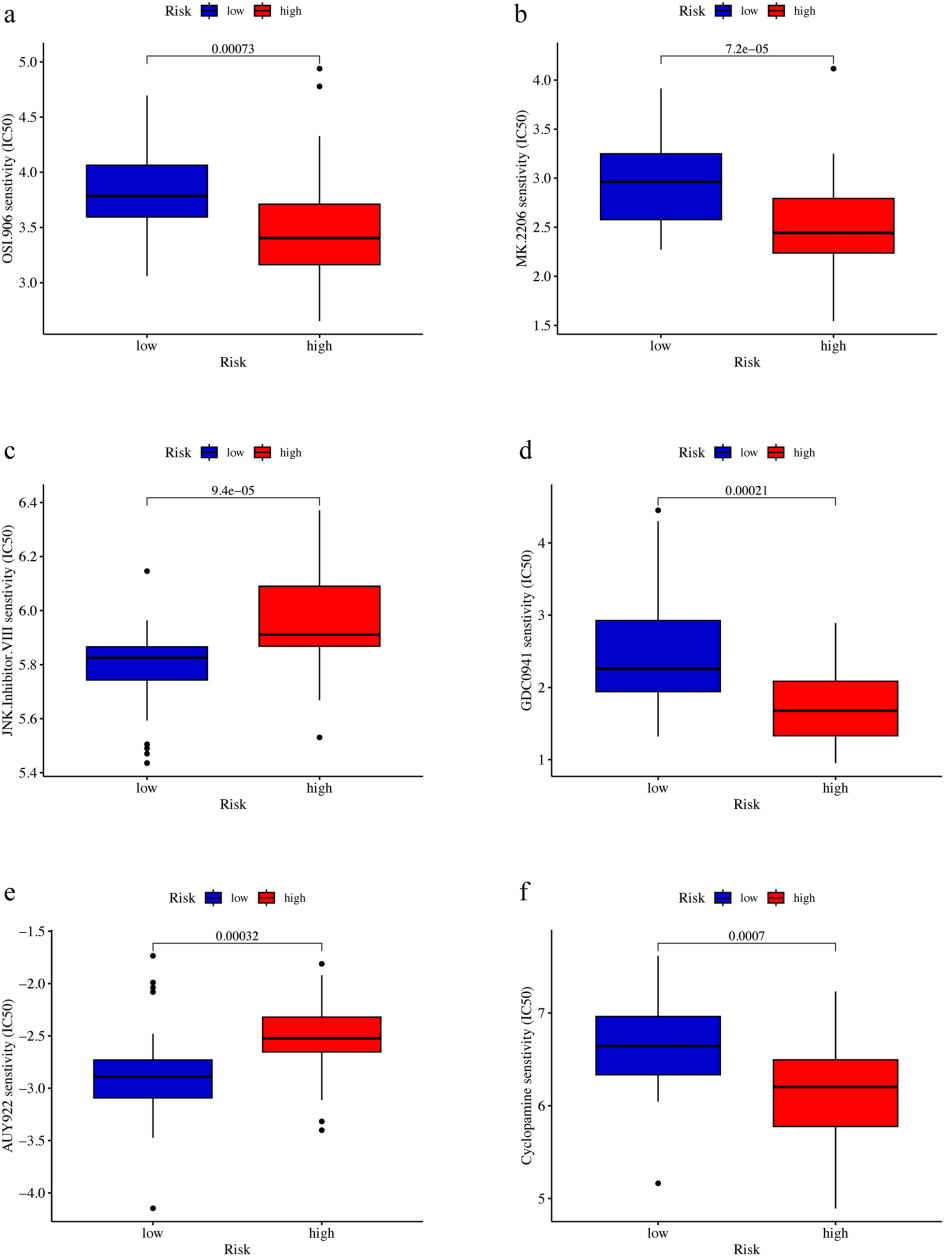
The box plots of IC50 differences of drugs in the high- and low-risk groups by drug sensitivity analysis

Six drugs were found to be significantly different in IC50 between the high- and low-risk groups (*p* < 0.05), implying a difference in response to these six drugs between the high- and low-risk groups. Specifically, linsitinib (OSI-906), AKT inhibitor (MK-2206), PI3K (Phosphatidylinositide 3-kinases) inhibitor (GDC0941) and cyclopamine were more effective in the high-risk group, while JNK Inhibitor VIII (TCS JNK 6o) and AUY922 were more effective in the low-risk group (Fig. 10).

## Discussion

Studies have shown that many characteristics of aging (such as epigenetic changes, changes in intracellular communication, changes in protein deposition, mitochondrial dysfunction and cell aging) are common to cancer (Aunan et al. 2017), which may be attributed to the fact that most cancers occur in the elderly (Hsu 2016). Although cellular senescence is a critical component of the aging process and an essential phenotype of tumor development, its role in juvenile osteosarcoma patients, who have not yet undergone senescence, remains unclear. Therefore, further study is needed to explore how cellular senescence functions in these particular patients and its potential implications for juvenile osteosarcoma development. Exploring the differences in tumor cell proliferation and cellular senescence between elderly and juvenile patients plays a vital role in understanding the underlying mechanisms of tumor occurrence and development. It also helps to comprehend the unique physical characteristics of individuals with different ages in relation to tumorigenesis. From the perspective of traditional Chinese medicine (TCM), the phenotype of juvenile is characterized by "immature Yin and Yang", "Yang is frequently in excess while Yin is often in deficiency", which often leads to an imbalance of "kidney Yang", consistent with the pathogenesis of osteosarcoma as understood in TCM. The present study will relate cellular senescence to kidney Yang and provide direction for subsequent experiments and clinical practice.

In this study, six cellular senescence-related lncRNAs with independent prognostic significance (*AL512330.1, SENCR, AC090559.1, LINC02710, AL022328.1* and *AC091271.1*) in juvenile osteosarcoma patients were screened by the univariate Cox regression analysis, LASSO regression analysis and multivariate Cox regression analyses. Subsequently, the survival analysis was used to further validate the prognostic value of the six lncRNAs, and a co-expression network between these six lncRNAs and the mRNAs were constructed. *SENCR* is closely associated with the proliferation and migration of vascular smooth muscle cells, and the polymorphic variants of *SENCR* affect the susceptibility and progression of Ewing sarcoma (Ye et al. 2020; Martinelli et al. 2022). *AL512330.1* and *AC090559.1* involve in lung adenocarcinoma and osteosarcoma by regulating autophagy, pyroptosis, ferroptosis and necroptosis (Guo et al. 2021; Wu et al. 2021; Liu et al. 2022b, 2022a). However, the role of *LINC02710*, *AL022328.1* and *AC091271.1* in cancer development has not been cleared and needs to be further investigated.

Here, we identified that the lncRNAs may have different roles in the progression of juvenile osteosarcoma. High expression levels of *AL512330.1*, *LINC02710*, AL022328.1 or *SENCR* were associated with short survival times, indicating the poor prognostic factors. In contrast, high expression level of *AC090559.1* or *AC091271.1* was associated with a long survival time, indicating a favorable prognostic factor. Overall, these results lay the groundwork for further molecular mechanism research and the development of new treatment strategies for juvenile osteosarcoma. By taking into account the multiple favorable and unfavorable prognostic factors will allow us to develop more comprehensive and personalized treatment regimens, as well as management plans to improve the quality and prolong the survival of juvenile osteosarcoma patients.

Based on our identified cellular senescence-related lncRNAs with independent prognostic significance, we further constructed a prognostic model in juvenile osteosarcoma patients. The patients were then divided into a high-risk group and a low-risk group based on the calculated median risk score of each sample. Furthermore, the prognostic model was validated by using the ROC curve, KM survival curve, distribution characteristics and gene expression heatmap, which showed that our prognostic model was reliable in revealing the prognosis of juvenile osteosarcoma patients.

Moreover, the downstream analysis was conducted for the cellular senescence-related lncRNAs with independent prognostic significance in juvenile osteosarcoma patients. The GO and KEGG enrichment analyses revealed that the cellular senescence-related lncRNAs were mainly involved in the occurrence and development of juvenile osteosarcoma by regulating lysosomes, proteasomes, peroxisomes, actin cytoskeletal race pathway, specific granules, tertiary granule membranes, and glycolipid binding. Studies have shown that lysosomes are crucial cell organelles in degradation, innate and adaptive immunity, as well as trophic sensing (Trivedi et al. 2020), which play important roles in tumor cell proliferation, invasion, radioresistance, and chemoresistance (Zhang et al. 2021). Dysregulation of lysosomal function is an important marker of cellular senescence and correlates with SA-β-gal secretion dysregulation in SASP (Lee et al. 2006). Proteasomes are the catalytic core of the ubiquitin proteasomal catalytic pathway (UPP), which regulates tumor cell growth, differentiation, drug resistance and apoptosis (Ignatz-Hoover et al. 2022). The autophagy-lysosome pathway and the UPP are the two main natural protein degradation pathways in cells and are closely associated with cellular senescence (N et al. 2020). Peroxisomes are organelles participate in lipid and reactive oxygen species (ROS) metabolism (Cipolla and Lodhi 2017). As cellular senescence, damaged proteins accumulate to form aggregates, which further exacerbated the cellular senescence, while peroxisomes slows down the aging by counteracting this damage mechanism (Baker et al. 2016). As one protein to make up the cytoskeleton, actin is the most abundant protein in eukaryotic cells and involved in more protein interactions than any other known proteins, playing an important role in cell morphology, cell motility, polarity and transcriptional regulation (Pollard and Cooper 2009; Dominguez and Holmes 2011; Biber et al. 2020). The actin cytoskeleton induces the abnormal protein expressions in many cancers to enhance the invasive and metastatic abilities of cancer cells (Hall 2009; Olson and Sahai 2009), while the defects in actin cytoskeleton are associated with cellular senescence (Moujaber et al. 2019). However, the specific granules, tertiary granule membranes and glycolipid binding have not been reported to participate the cellular senescence.

Most pathways identified through GO and KEGG analyses were intricately linked to both tumor development and cellular senescence, which may due to the essential components of SASP proteins, such as lysosomes, proteasomes, and peroxisomes, are closely interlinked with these pathways, further highlighting the importance of SASP in tumor development through cellular senescence. Moreover, it can be postulated that specific granules and tertiary granule membranes may be also involved in cellular senescence and contribute to tumorigenesis by modulating SASP. Granules are specialized vesicles storing bioactive molecules, and contribute to inflammation and tumorigenesis as key regulators of senescence associated cellular processes. In addition, tertiary granule membranes, formed by the fusion of primary and secondary granules, may also play a role in inflammation associated with senescence, thereby contributing to the pathogenesis of cancer.

By analyzing the immunological correlation, we further revealed that juvenile osteosarcoma patients in the low-risk group showed higher levels of the immune checkpoint expression, the immune function, and the immune cell infiltration than those in the high-risk group, indicating that patients in the low-risk group were more sensitive to immunotherapy, therefore, a better survival prognosis. These data further confirmed a relation between the cellular senescence and the immunity.

Meanwhile, m^6^A methylation modification has been reported to participate in osteosarcoma and cellular senescence development (Casella et al. 2019; Hu and Zhao 2021). However, we did not find significant differences in m^6^A-related genes between the high- and low-risk groups, and further investigation is necessary.

By drug sensitivity analysis, IC50 of the six drugs (linsitinib (OSI-906), AKT inhibitor MK-2206, PI3K inhibitor GDC0941, cyclopamine, JNK Inhibitor VIII (TCS JNK 6o) and AUY922) were found to be different in patients between the high- and low-risk groups. Among them, linsitinib (OSI-906), AKT inhibitor MK-2206, PI3K inhibitor GDC0941 and cyclopamine had lower IC50s in the high-risk group, implying that these drugs were more effective in the high-risk group. The other drugs (JNK inhibitor VIII (TCS JNK 6o) and AUY922) had higher IC50s in the high-risk group, implying that these drugs were less effective in the high-risk group. Many of these drugs (linsitinib (OSI-906), AKT inhibitor MK-2206, PI3K inhibitor GDC0941, Cyclopamine) have been shown to inhibit the progression of osteosarcoma (Warzecha et al. 2007; Hirai et al. 2010; Kuijjer et al. 2013; Wan et al. 2022), however their studies on the effect of treatment in the high- and low-risk groups based on cellular senescence-associated genes in juvenile osteosarcoma patients remain to be further carried out. The therapeutic effects of JNK Inhibitor VIII (TCS JNK 6o), PI3K inhibitor GDC0941 and AUY922 in osteosarcoma have not been clearly reported.

## Conclusion

Here, we investigated the relationship among juvenile osteosarcoma patients, cellular senescence and lncRNAs, which has been largely overlooked in previous researches. Firstly, osteosarcoma is more prevalent in juvenile, making it crucial to differentiate between disease populations; secondly, the unique physiological stage of juvenile seems to contradict 'senescence', thereby providing a better understanding of cellular senescence by examining whether it occurs in juvenile who have not yet undergone senescence; thirdly, this study combined cellular senescence with lncRNAs that were previously deemed 'noisy', and discovered that cellular senescence-associated lncRNAs possessed independent prognostic significance in the development of juvenile osteosarcoma. In addition, this study further explored the clinical significance of cellular senescence-related lncRNAs with independent prognostic significance, highlighting the potential implications for improving the prognosis of juvenile osteosarcoma patients and providing ideas for subsequent research concerning juvenile, cellular senescence and tumor correlation.

## Data Availability

The data and materials involved in the study were downloaded from the TCGA database (https://portal.gdc.cancer.gov/).

## Abbreviations

AUC: Area under the ROC curve
BP: Biological process
CC: Cellular component
CGP: Cancer Genome Project
CIBERSORT: Cell-type Identification By Estimating Relative Subsets of RNA Transcripts
CIBERSORT-ABS: Cell-type Identification By Estimating Relative Subsets of RNA Transcripts with Absolute Quantities
GO: Gene Ontology
GSEA: Gene Set Enrichment Analysis
IC50: Half-maximal inhibitory concentration
KEGG: Kyoto Encyclopedia of Genes and Genomes
KM: Kaplan–Meier
LASSO: Least absolute shrinkage and selection operator
LncRNA: Long non-coding RNA
MCP-counter: Microenvironment Cell Populations-counter
MiRNAs: MicroRNA
MF: Molecular function
NcRNAs: Non-coding RNAs
QuanTIseq: Quantifying immune contexture of human tumors
ROC: Receiver operating characteristic
ROS: Reactive oxygen species
SAβgal: Senescence-associated β-galactosidase
SASP: Senescence-associated secretory phenotype
SNCs: Senescent cells
TCGA: The Cancer Genome Atlas
TCM: Traditional Chinese medicine
TIMER: Tumor Immune Estimation Resource
